# A less selfish view of genome size evolution in maize

**DOI:** 10.1371/journal.pgen.1007249

**Published:** 2018-05-10

**Authors:** Julia M. Kreiner, Stephen I. Wright

**Affiliations:** Department of Ecology & Evolutionary Biology, University of Toronto, Toronto, Ontario, Canada; The University of North Carolina at Chapel Hill, UNITED STATES

Genome size varies by many orders of magnitude across plants and animals, but resolving the most important evolutionary forces driving this variation remains challenging. Since eukaryotic genome size variation is not associated with complexity, genetic drift of the amount of noncoding DNA could dominate, implicating population and species history as key drivers of shifts in DNA content. Alternatively, directional selection could be acting on DNA content, but if so, it has not been resolved which level of selection is most important. Since the predominant component of many eukaryotic genomes is comprised of selfish genetic elements such as transposable elements (TEs) and regions subject to meiotic drive, factors that influence their differential success across populations and species could account for much of the variation in genome size. However, DNA content can also have important effects on organismal phenotype that could be under directional selection. Genome size may often be an important determinant of cell size and division rate and the subject of selection via its effects on developmental and metabolic phenotypes [1,2]. Supporting this view, correlations of genome size with invasive potential and growth rate [3], regional abundance and seed size [4], and even metabolic intensity in flying birds [5], may suggest an adaptive role. In this issue, Bilinski et al. [6] present evidence for this organismal adaptation view of genome size evolution by providing an explicit test for natural selection on genome size and repeat abundance across multiple altitudinal clines in maize and its wild relatives and identifying the underlying physiological mechanism.

Bilinski et al. [6] capitalize on the remarkable genome size variation in maize and its wild relatives, which differ by 40%–70% within and between subspecies. It is estimated that 85% of the maize genome is composed of TEs, B chromosomes, and heterochromatic knobs subject to meiotic drive [7,8], highlighting the success of selfish genetic elements in this lineage. Nevertheless, clines of genome size along with phenotypic and environmental variables in *Zea mays* spp. have been well described, with a number of studies showing evidence of genome reductions, including the loss of knobs and B chromosomes, in regions of high altitude and latitude [9–13]. While these parallel clines are suggestive of an adaptive process, it has been difficult to definitively demonstrate this and fully reject a role for neutral population history.

Bilinski et al. [6] add to this rich literature by using a quantitative genetics framework to conduct a test of local adaptation, recognizing that genome size is a trait governed by an immense number of small-effect loci. Moreover, it can be thought of as a quantitative trait under complete genetic control, since the variation in genome size is expected to be a simple function of the net number of insertion and deletion alleles individuals have across the genome. Under this framework, a neutral model predicts that genome size differences across a geographic region are a function of the relatedness and population structure [14] and thus determined by the extent of correlated allele frequencies between a given pair of individuals. It follows that if genome size is subject to selection, it should be more strongly correlated with altitude than expected from relatedness alone. The authors use low-coverage whole genome sequence data from three altitudinal clines to obtain detailed information not only about the kinship and structure of their samples but also about the contribution of each type of repeat to overall genome size.

Using this approach to test for local adaptation, the authors are able to reject the predictions of the neutral model, concluding that genome size differences along altitudinal clines are too extreme to be explained solely by drift (Fig 1). When considering individual repeat types, both TEs and knob repeat abundance are significantly correlated with altitude. They also find that one type of knob repeat, known as TR1, shows the strongest over-differentiation, and the signal of altitudinal adaptation on TR1 abundance remains significant even when controlling for genome size. With megabase-long heterochromatic knobs accounting for up to 10% of maize genome size variation [7,15], knobs seem to be acting as large-effect loci for genome size but, in some cases, may also be under additional selection pressures independent of their effects on genome size. Although TEs do not show signals of adaptation after controlling for genome size, the strong correlation of TE copy number with genome size and altitude implies that environmental adaptation could also be an important determinant of TE abundance mediated through its effects on genome size.

**Fig 1:**
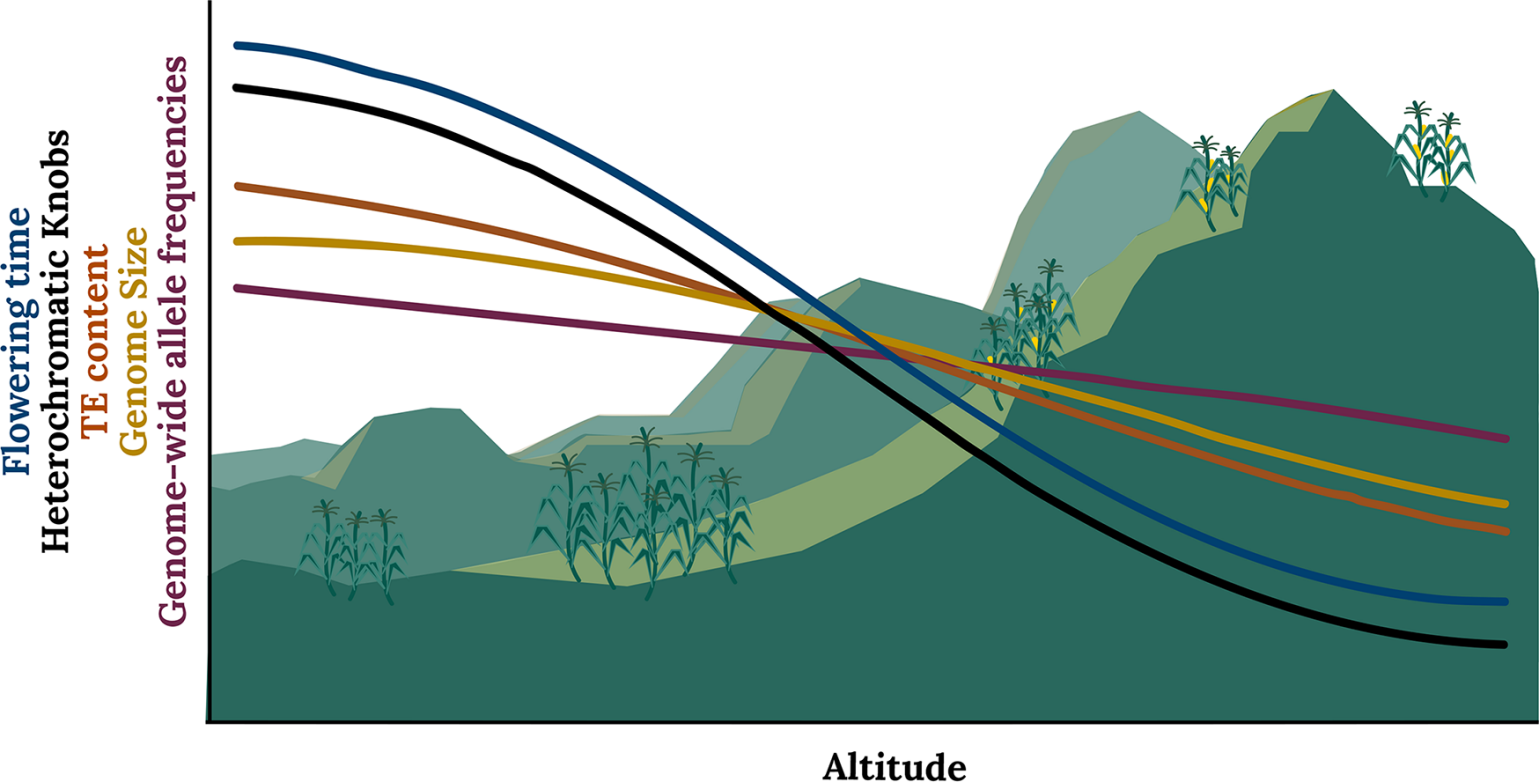
Altitudinal clines in maize and teosinte. Clines in heterochromatic knobs and genome size in *Zea mays* spp. are significantly over-dispersed compared to genome-wide allele frequencies, corresponding with an adaptive response to selection for earlier flowering time at higher altitudes. TE content is also a major determinant of genome size variation and thus also covaries significantly with altitude. TE, transposable element.

With the finding that natural selection shapes genome size variation across multiple parallel altitudinal clines in maize and its wild relatives, Bilinski et al. [6] searched for the phenotypic target of selection. It has been proposed that genome size evolution may be mediated by its effects on flowering time [10,13,16], given that populations at higher latitudes are typically early-flowering [17,18], and a previous selection experiment on flowering time led to a correlated decrease in genome size [16]. This could result from the strong scaling between genome size, cell size, and cell cycle length [19]. Bilinski et al. [6] were able to uncouple a role for population history driving an indirect association between genome size and these traits through performing a growth chamber experiment on a single highly variable population and controlling for family structure. They then infer a causal relationship between genome size, cell production rate, and flowering time through a path analysis. This mechanistic connection, combined with the body of past work, provides a compelling case for flowering time as the driver of altitudinal genome downsizing with large chromosomal knobs being a major factor under selection. A parallel study in maize identified the region with the most explanatory power for genome size to map near two 180-bp knobs where genome size was also correlated with flowering time, but only before controlling for relatedness—perhaps due to greater effects of population structure in their study [20]. Although the fraction of flowering time variation controlled by genome size is still unclear, Bilinski et al. [6] add to growing evidence for the case of genome size evolution driven by phenotypic selection, in line with a recent large-scale phylogenetic study that argues genome downsizing was the key to angiosperms’ higher photosynthetic efficiency [21].

This paper provides important evidence for the organismal-adaptation view of genome size evolution—but what of the coevolutionary struggle with selfish elements? The major evolutionary force driving genome expansion in maize is still likely to reflect selfish evolution through selection on meiotic drivers and self-replicating TEs, since selection pressures on such alleles are typically very strong. Knob elements, for example, have a strong transmission advantage driving their increase [22]. On the other hand, the factors stabilizing unregulated genome expansion or driving genome shrinkage may be multifaceted. The explosion of selfish genetic elements is typically thought to be controlled by unconditionally deleterious effects such as meiotic defects on pollen viability and seed set [22], ectopic recombination events causing chromosomal rearrangements [23–25], and the harmful effects of gene disruption. However, the results found by Bilinski et al. [6] highlight that some costs to selfish genetic elements may be environmentally dependent, determined by the indirect effects of repetitive elements on flowering time through their effects on genome size. In regions of high elevation, stronger selection against large genomes could lead to greater control of total repetitive element abundance. For large-effect alleles such as chromosomal knobs, such environmentally dependent selection could explain why their population frequencies are somewhat lower than expected by their effects on pollen viability and seed set alone [22].

Conversely, other selective pressures on selfish genetic elements may provide an additional source of genome size variation in maize populations beyond the effects of flowering time. Since chromosomal knobs are known to have severe fitness costs, the extent of these costs may well depend on the environment [22], possibly explaining the disproportionate effect of altitude on knobs when genome size is controlled for in this study. For instance, if conditions are more extreme at higher elevations, the costs of knobs on fecundity may be felt more severely in the absence of buffering from favorable conditions. The costs of TEs and heterochromatic knobs may also be more severe in inbred genomic backgrounds for example; since knobs have recessive deleterious costs [22], they should be subject to strong purifying selection when homozygous. Consistent with this, inbred lines of domesticated maize have been found to be mostly absent of knobs [26]. This may explain why the smallest genomes in the present study were found in the three lowest-altitude teosinte populations, providing an exception to the observed altitudinal gradient. These populations are known to be highly inbred, although admixture from related species may also be playing a role. Genome size variation in maize may thus result from the dynamic interplay between the selfish spread of knobs and TEs, heterogeneity in their costs and the efficacy of selection against them, and the exaptation of the resulting variation for the optimization of developmental traits such as flowering time. This suggests a pluralistic model whereby selfish evolution, environmental adaptation, and population history all contribute to evolutionary fluctuations in DNA content.

## References

[1] Commoner B. Roles of deoxyribonucleic acid in inheritance. Nature. 1964;202: 960–968. 14197326 10.1038/202960a0

[2] Bennett MD. Nuclear DNA content and minimum generation time in herbaceous plants. Proc R Soc Lond B Biol Sci. 1972;181: 109–135. 4403285 10.1098/rspb.1972.0042

[3] Lavergne S, Muenke NJ, Molofsky J. Genome size reduction can trigger rapid phenotypic evolution in invasive plants. Ann Bot. 2010;105: 109–116. doi: 10.1093/aob/mcp271 19887472 10.1093/aob/mcp271 PMC2794072

[4] Herben T, Suda J, Klimesová J, Mihulka S, Ríha P, Símová I. Ecological effects of cell-level processes: genome size, functional traits and regional abundance of herbaceous plant species. Ann Bot. 2012;110: 1357–1367. doi: 10.1093/aob/mcs099 22628380 10.1093/aob/mcs099 PMC3489144

[5] Wright NA, Gregory TR, Witt CC. Metabolic “engines” of flight drive genome size reduction in birds. Proc Biol Sci. 2014;281: 20132780 doi: 10.1098/rspb.2013.2780 24478299 10.1098/rspb.2013.2780 PMC3924074

[6] Bilinski P, Albert PS, Berg JJ, Birchler JA, Grote M, Lorant A, et al (2018) Parallel altitudinal clines reveal trends in adaptive evolution of genome size in *Zea mays*. PLoS Genet 14(3): e1007249 10.1371/journal.pgen.1007162 PMC5944917 29746459

[7] Tenaillon MI, Hufford MB, Gaut BS, Ross-Ibarra J. Genome size and transposable element content as determined by high-throughput sequencing in maize and Zea luxurians. Genome Biol Evol. 2011;3: 219–229. doi: 10.1093/gbe/evr008 21296765 10.1093/gbe/evr008 PMC3068001

[8] Schnable PS, Ware D, Fulton RS, Stein JC, Wei F, Pasternak S, et al The B73 maize genome: complexity, diversity, and dynamics. Science. 2009;326: 1112–1115. doi: 10.1126/science.1178534 19965430 10.1126/science.1178534

[9] Bretting PK, Goodman MM. Karyotypic variation in Mesoamerican races of maize and its systematic significance. Econ Bot. Springer-Verlag; 1989;43: 107–124.

[10] Poggio L, Rosato M, Chiavarino AM, Naranjo CA. Genome Size and Environmental Correlations in Maize (Zea mays ssp. mays, Poaceae). Ann Bot. Oxford University Press; 1998;82: 107–115.

[11] Rosato M, Chiavarino A, Naranjo C, Hernandez J, Poggio L. Genome size and numerical polymorphism for the B chromosome in races of maize (Zea mays ssp. mays, Poaceae). Am J Bot. 1998;85: 168 21684902

[12] Buckler ES 4th, Phelps-Durr TL, Buckler CS, Dawe RK, Doebley JF, Holtsford TP. Meiotic drive of chromosomal knobs reshaped the maize genome. Genetics. 1999;153: 415–426. 10471723 10.1093/genetics/153.1.415 PMC1460728

[13] Díez CM, Gaut BS, Meca E, Scheinvar E, Montes-Hernandez S, Eguiarte LE, et al Genome size variation in wild and cultivated maize along altitudinal gradients. New Phytol. 2013;199: 264–276. doi: 10.1111/nph.12247 23550586 10.1111/nph.12247 PMC4119021

[14] Berg JJ, Coop G. A Population Genetic Signal of Polygenic Adaptation. PLoS Genet. Public Library of Science; 2014;10: e1004412 doi: 10.1371/journal.pgen.1004412 25102153 10.1371/journal.pgen.1004412 PMC4125079

[15] Ananiev EV, Phillips RL, Rines HW. A knob-associated tandem repeat in maize capable of forming fold-back DNA segments: are chromosome knobs megatransposons? Proc Natl Acad Sci U S A. 1998;95: 10785–10790. 9724782 10.1073/pnas.95.18.10785 PMC27973

[16] Rayburn AL, Dudley JW, Biradar DP. Selection for Early Flowering Results in Simultaneous Selection for Reduced Nuclear DNA Content in Maize. Plant Breed. Blackwell Publishing Ltd; 1994;112: 318–322.

[17] Jiang C, Edmeades GO, Armstead I, Lafitte HR, Hayward MD, Hoisington D. Genetic analysis of adaptation differences between highland and lowland tropical maize using molecular markers. Theor Appl Genet. Springer-Verlag; 1999;99: 1106–1119.

[18] Rodriguez JG, Sánchez G, Baltazar BM, De la Cruz LL, Santacruz-Ruvalcaba F, Ron PJ, et al Characterization of floral morphology and synchrony among Zea species in Mexico. Maydica. 2006; Available: http://agris.fao.org/agris-search/search.do?recordID=IT2007602235

[19] Símová I, Herben T. Geometrical constraints in the scaling relationships between genome size, cell size and cell cycle length in herbaceous plants. Proc Biol Sci. 2012;279: 867–875. doi: 10.1098/rspb.2011.1284 21881135 10.1098/rspb.2011.1284 PMC3259922

[20] Jian Y, Xu C, Guo Z, Wang S, Xu Y, Zou C. Maize (Zea mays L.) genome size indicated by 180-bp knob abundance is associated with flowering time. Sci Rep. 2017;7: 5954 doi: 10.1038/s41598-017-06153-8 28729714 10.1038/s41598-017-06153-8 PMC5519714

[21] Simonin KA, Roddy AB. Genome downsizing, physiological novelty, and the global dominance of flowering plants. PLoS Biol. 2018;16: e2003706 doi: 10.1371/journal.pbio.2003706 29324757 10.1371/journal.pbio.2003706 PMC5764239

[22] Higgins DM, Lowry EG, Kanizay LB, Becraft PW, Hall DW, Dawe RK. Fitness Costs and Variation in Transmission Distortion Associated with the Abnormal Chromosome 10 Meiotic Drive System in Maize. Genetics. 2018;208: 297–305. doi: 10.1534/genetics.117.300060 29122827 10.1534/genetics.117.300060 PMC5753864

[23] Kupiec M, Petes TD. Allelic and ectopic recombination between Ty elements in yeast. Genetics. 1988;119: 549–559. 2841187 10.1093/genetics/119.3.549 PMC1203441

[24] Han K, Lee J, Meyer TJ, Remedios P, Goodwin L, Batzer MA. L1 recombination-associated deletions generate human genomic variation. Proc Natl Acad Sci U S A. 2008;105: 19366–19371. doi: 10.1073/pnas.0807866105 19036926 10.1073/pnas.0807866105 PMC2614767

[25] Delprat A, Negre B, Puig M, Ruiz A. The transposon Galileo generates natural chromosomal inversions in Drosophila by ectopic recombination. PLoS ONE. 2009;4: e7883 doi: 10.1371/journal.pone.0007883 19936241 10.1371/journal.pone.0007883 PMC2775673

[26] Chia J-M, Song C, Bradbury PJ, Costich D, de Leon N, Doebley J, et al Maize HapMap2 identifies extant variation from a genome in flux. Nat Genet. 2012;44: 803–807. doi: 10.1038/ng.2313 22660545 10.1038/ng.2313

